# Association between estimated glomerular filtration rate (eGFR) and asymmetric dimethylarginine (ADMA) concentrations among the elderly in a rural community: a cross-sectional study

**DOI:** 10.1186/s12877-019-1388-4

**Published:** 2019-12-23

**Authors:** Hye Rin Choi, Seung Won Lee, Da-Hye Jeon, Nam Wook Hur, Yoosik Youm, Hyeon Chang Kim

**Affiliations:** 1 Department of Public Health, Yonsei University of Graduate School, Seoul, Republic of Korea; 2 Cardiovascular and Metabolic Diseases Etiology Research Center, Seoul, Republic of Korea; 3 Department of Preventive Medicine, Yonsei University College of Medicine, Korea, Seoul, Republic of Korea; 4 Department of Sociology, Yonsei University College of Sociology, Seoul, Republic of Korea

**Keywords:** Glomerular filtration rate, Endothelium, vascular, Aged, Rural population

## Abstract

**Background:**

Reduced glomerular filtration rate and increased asymmetric dimethylarginine (ADMA) are prevalent in elderly people. However, most of the studies that have examined the association between the two conditions were performed in patients with renal dysfunction, but not in the general elderly population. Thus, we investigated an association between estimated glomerular filtration rate (eGFR) and ADMA concentration among community-dwelling older Koreans.

**Methods:**

A cross-sectional study was conducted on 269 men and 382 women (mean age, 71.6 years) enrolled in the Korean Social Life, Health, and Aging Project (KSHAP), a population-based cohort study of health determinants in elderly Koreans. We calculated eGFR using chronic kidney disease- Epidemiology Collaboration Group (CKD-EPI) equation. ADMA concentration was measured by an enzyme-linked immunosorbent assay. The association between eGFR and ADMA concentrations was analyzed by multiple linear regression models.

**Results:**

The mean ADMA was significantly higher in people with eGFR< 60 mL/min/1.73m^2^ (0.691 μmol/L) than in those with eGFR≥60 mL/min/1.73m^2^ (0.667 μmol/L, *p* = 0.013). The negative correlations between eGFR level and ADMA concentrations were significant in men and women after adjusted age. After adjusting for potential confounders which were sex, age, body surface, blood pressure, total and HDL cholesterol, diabetes, smoking, and drinking, eGFR levels were inversely associated with ADMA concentrations both in men (β = − 0.0015, *p* = 0.005) and women (β = − 0.001, *p* = 0.039).

**Conclusion:**

Our findings suggest that an inverse association exists between eGFR and ADMA concentrations among the Korean elderly in a rural community.

## Background

The prevalence of chronic kidney disease has been increasing among elderly people, because aging affects kidney function and arterial stiffness [1, 2]. As the elasticity of the blood vessels decreases, the surrounding blood vessels cannot protect the cells of the kidneys [3]. Asymmetric dimethylarginine (ADMA) is an endogenous nitric oxide (NO) synthase inhibitor [4]. ADMA is eliminated through renal excretion and is metabolized by a dimethylarginine dimethylaminohydrolase (DDAH), which is found in the tissues of the kidney, pancreas, and blood vessels [5]. Therefore, the kidney plays an important role in maintaining reduced plasma ADMA concentrations. However, among elderly people, DDAH secretion is decreased and ADMA concentration is increased because of reduced kidney function due to aging. Inhibiting NO synthase impairs endothelium-dependent vasodilation, resulting in endothelial dysfunction [6]. Based on previous studies, we hypothesized that reduced eGFR would elevate plasma ADMA concentrations in elderly people. However, most of the previous papers were conducted only for renal patients, and the relationship between eGFR and ADMA concentrations in the general healthy population has not been rigorously studied. Hence, we investigate the association between eGFR and plasma ADMA concentrations among the elderly in a rural community.

## Methods

### Study population

Data for this study was collected from the Korean Social Life, Health, and Aging Project (KSHAP) cohort study, which started in 2011. The KSHAP study recruited individuals aged 60 years or older and their spouses living in Township K located on Ganghwa Island, South Korea. As of January 2013, the total population who lived in Township K was estimated at 1864 people and 871 families. With the aid of township officers and after performing a pilot study, a total of 860 people who aged over 60 years and their spouses was identified as the target population of KSHAP. To obtain their consents and perform questionnaires, we visited participants’ homes individually. A total of 814 of the 860 community-dwelling adults (response rate, 94.7%) participated in the study and finished the questionnaire surveys between December 2011 and July 2012 [7]. Among them, 698 people completed the KSHAP-Health Examination at a public health center (*n* = 533) or at home (*n* = 165) [8]. In this paper, 47 were excluded for missing key variables, such as eGFR (*n* = 23), plasma ADMA concentration *n* = 19), and body mass index (BMI) (n = 5), leaving 651 people (269 men and 382 women) for this analysis. All participants provided written informed consent.

### Measurements

Our trained personnel interviewed participants using standardized questionnaire surveys according to a predefined protocol. We obtained individuals’ socio-demographic characteristics including age, education, occupation, religion, economic and marital status, smoking and drinking habits, and medical history. Further detailed explanation for this questionnaire was published in the cohort profile paper [9].

Standing height was measured to the nearest 0.1 cm with a stadiometer. We measured body weight to the nearest 0.1 kg with a digital scale according to the predefined manual. BMI was calculated as an individual’s body weight divided by height squared (kg/m^2^). Body surface area (BSA) was calculated by a formula; [{(weight) x (height)}/3600]^1/2^. We measured blood pressure for two times using an automatic sphygmomanometer (Dinamap 1846 SX/P; GE Healthcare, Waukesha, WI, USA) after participants rested for at least five minutes in a seated position. If the two measurements differed by 10 mmHg or more, additional measurements were performed after five minutes [10]. The average of the last two measurements was used in this study.

**Fig. 1:**
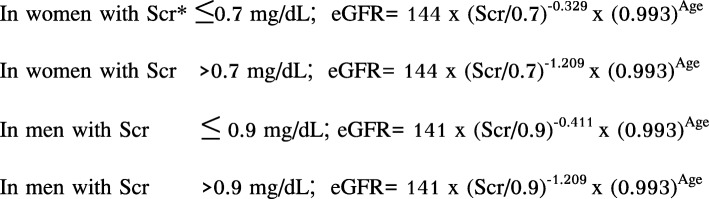
CKD-EPI equations for women and men

Individuals’ blood samples were collected after at least eight hours’ fasting. Serum creatinine concentrations were analyzed using a colorimetric Jaffe, Alkaline picrate, kinetic method (ADVIA1800 Auto Analyzer, Siemens Medical Sol., Deerfield, IL, USA). Concentrations of blood urea nitrogen and glucose were analyzed using a colorimetry-based method (ADVIA1800 Auto Analyzer, Siemens Medical Sol., Deerfield, IL, USA). Fasting insulin concentration was measured by using an immunoradiometric assay (SR-300, Stratec, Germany). Total cholesterol, high-density lipoprotein (HDL) cholesterol, and triglyceride levels were assayed by enzymatic methods (ADVIA1800 Auto Analyzer, Siemens Medical Sol., Deerfield, IL, USA). Participants’ eGFR was calculated by chronic kidney disease- Epidemiology Collaboration Group (CKD-EPI) equation, which was developed and validated in 2009 to predict CKD more accurately in people with eGFR > 60 ml/min/1.73m^2^ [11, 12]. The CKD-EPI formula was shown in Fig. 1. Furthermore, all participants were classified into two eGFR groups according to National Kidney Foundation criteria: normal to minimally-reduced eGFR (≥60 ml/min /1.73m^2^) and moderately to severely-reduced eGFR (< 60 ml/min /1.73m^2^) [13]. In addition, ADMA concentration was measured by an enzyme-linked immunosorbent assay (Spectramax190, Molecular Devices, USA). We decided that ADMA elevation corresponds to a concentration above the 75th percentile. Hypertension was defined as systolic blood pressure (SBP) ≥140 mmHg, diastolic blood pressure (DBP) ≥90 mmHg, or current use of anti-hypertensive medicine. Diabetes was defined as fasting glucose ≥126 mg/dL or current use of oral anti-diabetic medicine or insulin. Hypercholesterolemia was defined as total cholesterol ≥240 mg/dL, HDL cholesterol < 40 mg/dL or current treatment by anti-hyperlipidemic agents.

### Statistical analysis

Sex differences of baseline characteristics were analyzed by t-test for continuous variables and Chi-square test for categorical variables. We also compared participants’ characteristics between groups of eGFR < 60 and ≥ 60 mL/min/1.73m^2^. Differences in general characteristics among two groups were analyzed using t-test for continuous variables and Chi-square test for categorical variables. Continuous variables that followed a normal distribution are shown as mean and standard deviation, whereas skewed variables are expressed as median and interquartile range. Categorical variables were described as numbers and percentages. Fasting glucose and insulin, triglycerides, serum creatinine, and blood urea nitrogen were log-transformed for parametric analysis due to the right-skewed distributions.

Correlation between eGFR and ADMA concentrations was assessed by Spearman’s coefficients in men and women individually, because eGFR level was skewed to right. To evaluate independent associations between continuous eGFR and plasma ADMA concentration, we carried out multiple linear regression analyses in three adjusted models: model 1 was unadjusted analyses; model 2 was adjusted for blood pressure, diabetes, total cholesterol, HDL cholesterol, smoking status, and drinking status; and model 3 was adjusted for sex, age, BMI, blood pressure, diabetes, total cholesterol, HDL cholesterol, smoking status, and drinking status.

All analyses were performed with SAS version 9.4 (SAS Institute, Cary, NC, USA), and statistical significance was defined as a two-sided *p*-value less than 0.05.

## Results

Differences of baseline characteristics in total, men, and women are described in Table 1. A total of 651 participants (269 men and 382 women) were included in this study. The distributions of eGFR < 60 mL/min/1.73m^2^ were 21.6% in men and 30.4% in women. The mean age of men and women were 72.2 and 71.1 years old. Men had significantly higher BSA, eGFR level, BUN, serum creatinine concentrations, DBP, fasting glucose level, and higher frequency of current smoker and drinker, compared with women. On the other hand, the mean of BMI, pulse pressure, fasting insulin, total cholesterol, and triglycerides were significantly higher in women than men. However, there were no significant differences of ADMA concentrations between men and women.

**Table 1:** General characteristics of study population in total, men, and women

Variables	Total (*n* = 651)			Men (*n* = 269)			Women (*n* = 382)			*p*-value
Age, yr	71.6	±	7.5	72.2	±	6.8	71.1	±	7.9	0.061
Body mass index, kg/m^2^	24.0	±	3.4	23.6	±	3.4	24.3	±	3.4	0.007
Body surface area	1.3	±	0.3	1.4	±	0.3	1.1	±	0.2	< 0.001
eGFR, mL/min/1.73m^2^	67.7	[59.5, 76.2]		69.4	[61.0, 77.5]		65.9	[57.9, 74.3]		0.003
eGFR< 60	174	(26.7)		58	(21.6)		116	(30.4)		0.012
eGFR≥60	477	(73.3)		211	(78.4)		266	(69.6)		
BUN, mg/dL	15.8	[13.1, 19.1]		16.6	[13.9, 19.3]		15.2	[12.7, 18.7]		0.001
Creatinine, mg/dL	0.95	[0.85, 1.09]		1.06	[0.97, 1.19]		0.88	[0.81, 0.98]		< 0.001
ADMA, μmol/L	0.674	±	0.111	0.677	±	0.109	0.671	±	0.112	0.542
ADMA elevation *	182	(28.0)		80	(12.3)		102	(15.7)		0.396
Systolic BP, mmHg	134.2	±	20.1	132.9	±	19.8	135.1	±	20.3	0.165
Diastolic BP, mmHg	72	±	10.1	73.1	±	10.4	71.2	±	9.9	0.023
Pulse pressure, bpm	62.2	±	16.9	59.9	±	15.8	63.9	±	17.5	0.003
Fasting glucose, mg/dL	90	[83, 101]		91	[83, 106]		89	[83, 98]		0.034
Fasting insulin, uIU/mL	7.6	[5.9, 10.3]		7.0	[5.5, 10.4]		7.9	[6.2, 10.2]		0.006
Total cholesterol, mg/dL	181.9	±	36	172.2	±	34.6	188.7	±	35.5	< 0.001
HDL cholesterol, mg/dL	50.9	±	12.7	50.1	±	13.3	51.5	±	12.3	0.147
Triglycerides, mg/dL	136	[102, 188]		129	[94, 178]		141	[105, 193]		0.029
Diabetes, %	150	(23.0)		71	(26.4)		79	(20.7)		0.090
Hypertension, %	420	(64.5)		163	(60.6)		257	(67.3)		0.080
Hyperlipidemia, %	181	(27.8)		75	(27.9)		106	(27.8)		0.970
Smoking										
Non-smoker	456	(70.0)		82	(30.5)		374	(97.9)		< 0.001
Past smoker	117	(18.0)		117	(43.5)		0	(0.0)		
Current smoker	78	(12.0)		70	(26.0)		8	(2.1)		
Drinking										
Non-drinker	512	(78.7)		154	(57.3)		358	(93.7)		< 0.001
Current drinker	139	(21.3)		115	(42.7)		24	(6.3)		

Values are shown as mean ± SD, median [IQR], or number (%)

eGFR, estimated glomerular filtration rate; BP, blood pressure; LDL, low-density lipoprotein; HDL, high-density lipoprotein; BUN, blood urea nitrogen; ADMA, asymmetric dimethylarginine

* ADMA elevation, people with ADMA concentrations >75th percentile

Table 2 shows general characteristics of the study participants according to eGFR groups; eGFR< 60 and ≥ 60 mL/min/1.73m^2^. The mean ADMA concentration was significantly higher in people with eGFR < 60 mL/min/1.73m^2^ (0.691 μmol/L, *p* = 0.013) compared to those with eGFR ≥60 mL/min/1.73m^2^ (0.667 μmol/L). Participants with lower eGFR had significantly older age, higher BUN, creatinine, SBP, pulse pressure, fasting glucose and insulin level compared to those with higher eGFR. The prevalence of diabetes and hypertension were significantly higher in group of eGFR < 60 mL/min/1.73m^2^.

**Table 2:** General characteristics in groups of eGFR< 60 and ≥ 60 mL/min/1.73m^2^

Variables	Total (n = 651)			eGFR< 60 (*n* = 174)			eGFR≥60 (*n* = 477)			*p*-value
Age, yr	71.6	±	7.5	75.3	±	7.8	70.1	±	6.8	< 0.001
Body mass index, kg/m^2^	24.0	±	3.4	23.7	±	3.5	24.1	±	3.4	0.247
Body surface area	1.3	±	0.3	1.2	±	0.3	1.3	±	0.3	< 0.001
eGFR, mL/min/1.73m^2^	67.7	[59.5, 76.2]		53.0	[45.4, 57.0]		71.4	[66.2, 78.0]		< 0.001
BUN, mg/dL	15.8	[13.1, 19.1]		18.1	[15.4, 23.3]		15.0	[12.6, 17.8]		< 0.001
Creatinine, mg/dL	0.95	[0.85, 1.09]		1.17	[1.00, 1.34]		0.90	[0.83, 1.01]		< 0.001
ADMA, μmol/L	0.674	±	0.111	0.691	±	0.112	0.667	±	0.11	0.013
ADMA elevation *	182	(28.0)		57	(31.3)		125	(26.7)		0.237
Systolic BP, mmHg	134.2	±	20.1	137.3	±	21.6	133	±	19.4	0.014
Diastolic BP, mmHg	72	±	10.1	71.1	±	10.5	72.3	±	10	0.172
Pulse pressure, bpm	62.2	±	16.9	67.2	±	18.1	61.8	±	14.2	0.003
Fasting glucose, mg/dL	90	[83, 101]		91	[84, 107]		89	[83, 99]		0.001
Fasting insulin, uIU/mL	7.6	[5.9, 10.3]		8.4	[6.2, 13.2]		7.4	[5.8, 9.9]		0.002
Total cholesterol, mg/dL	181.9	±	36	175.3	±	38.7	184.4	±	34.7	0.003
HDL cholesterol, mg/dL	50.9	±	12.7	48.6	±	12.5	51.8	±	12.7	0.004
Triglycerides, mg/dL	136	[102, 188]		149	[109, 193]		129	[100, 185]		0.844
Diabetes, %	150	(23.0)		55	(30.2)		95	(20.3)		0.008
Hypertension, %	420	(64.5)		139	(76.4)		281	(59.9)		< 0.001
Hyperlipidemia, %	181	(27.8)		57	(31.3)		124	(26.4)		0.216
Smoking										
Non-smoker	456	(70.0)		131	(72.0)		325	(69.3)		0.792
Past smoker	117	(18.0)		31	(17.0)		86	(18.3)		
Current smoker	78	(12.0)		20	(11.0)		58	(12.4)		
Drinking										
Non-drinker	512	(78.7)		157	(86.3)		355	(75.7)		0.002
Current drinker	139	(21.3)		25	(13.7)		114	(24.3)		

Values are shown as mean ± SD, median [IQR], or number (%)

eGFR, estimated glomerular filtration rate; BP, blood pressure; LDL, low-density lipoprotein; HDL, high-density lipoprotein; BUN, blood urea nitrogen; ADMA, asymmetric dimethylarginine

* ADMA elevation, people with ADMA concentrations >75th percentile

**Fig. 2:**
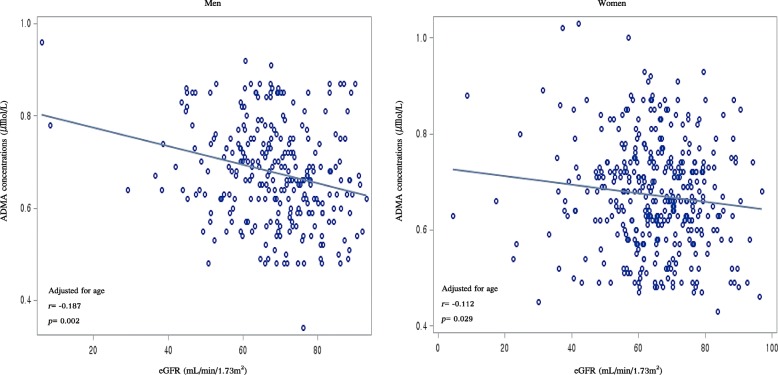
Spearman’s correlations between eGFR levels and plasma ADMA concentrations

Figure 2 presents the correlation of eGFR and plasma ADMA concentrations in men and women by using Spearman’s coefficients with scatter plots. The eGFR was negatively correlated with ADMA concentrations among men in the unadjusted and age-adjusted models. In women, the inverse correlation between eGFR and ADMA concentrations was significant in age-adjusted model.

Table 3 outlines the linear association of continuous eGFR and ADMA concentrations from multiple linear regression analyses in total and groups stratified by eGFR level. Among total participants, the negative association of eGFR and ADMA concentration was significant in an unadjusted model. After fully adjusting for sex, age, BSA, blood pressure, total and HDL cholesterol, diabetes, smoking status, and alcohol consumption, the eGFR was significantly and inversely associated with ADMA concentration. In people with eGFR ≥60 mL/min/1.73m^2^, low eGFR was significantly associated with high ADMA concentrations before and after multiple adjusted. Although there were no statistical significant association of eGFR with ADMA concentrations among those with eGFR < 60 mL/min/1.73m^2^, the negative associations were also presented before and after fully adjusted for confounders.

**Table 3:** Association between eGFR and ADMA concentrations in total participants groups stratified by eGFR level

Variables	model 1		model 2		model 3	
	β (μmol/L)	*p* value	β (μmol/L)	*p* value	β (μmol/L)	*p* value
Total participants						
eGFR (mL/min/1.73m^2^)	−0.0013	<.0001	−0.0013	0.000	−0.0013	0.000
Systolic BP (mmHg)			− 0.0001	0.766	−0.0001	0.768
Diastolic BP (mmHg)			−0.0001	0.872	−0.0001	0.868
Total cholesterol (mg/dL)			0.0000	0.896	0.0000	0.802
HDL cholesterol (mg/dL)			0.0000	0.952	0.0000	0.997
Diabetes Mellitus			−0.0017	0.870	−0.0022	0.839
Current smokers (for non-smokers)			0.0221	0.001	0.0245	0.004
Current drinkers (for non-drinkers)			− 0.0227	0.055	−0.0218	0.074
Sex					0.0075	0.595
Age (yr)					0.0001	0.912
BSA					0.0071	0.734
eGFR ≥ 60 (*n* = 477)						
eGFR (mL/min/1.73m^2^)	−0.0017	0.006	− 0.0017	0.005	− 0.0017	0.008
Systolic BP (mmHg)			−0.0004	0.263	−0.0003	0.304
Diastolic BP (mmHg)			0.0000	0.979	−0.0001	0.917
Total cholesterol (mg/dL)			0.0000	0.892	0.0000	0.952
HDL cholesterol (mg/dL)			−0.0001	0.812	−0.0001	0.903
Diabetes Mellitus			0.0075	0.552	0.0070	0.584
Current smokers (for non-smokers)			0.0211	0.008	0.0220	0.023
Current drinkers (for non-drinkers)			−0.0257	0.052	−0.0263	0.057
Sex					0.0040	0.808
Age (yr)					0.0000	0.959
BSA					0.0111	0.646
eGFR < 60 (*n* = 174)						
eGFR (mL/min/1.73m^2^)	−0.0012	0.143	−0.0012	0.148	−0.0012	0.164
Systolic BP (mmHg)			0.0005	0.280	0.0005	0.319
Diastolic BP (mmHg)			0.0001	0.882	0.0001	0.948
Total cholesterol (mg/dL)			−0.0001	0.550	−0.0002	0.533
HDL cholesterol (mg/dL)			0.0002	0.801	0.0002	0.825
Diabetes Mellitus			−0.0226	0.244	−0.0219	0.292
Current smokers (for non-smokers)			0.0193	0.172	0.0235	0.205
Current drinkers (for non-drinkers)			−0.0061	0.824	−0.0052	0.854
Sex					0.0093	0.761
Age (yr)					−0.0001	0.943
BSA					0.0004	0.992

eGFR, estimated glomerular filtration rate; BP, blood pressure; HDL, high-density lipoprotein; BSA, body surface area

Model 1: unadjusted

Model 2: adjusted for blood pressure, total and HDL cholesterol, diabetes mellitus, smoking status, and drinking status

Model 3: adjusted for sex, age, BSA, blood pressure, total and HDL cholesterol, diabetes mellitus, smoking status, and drinking status

Table 4 also describes relationships between eGFR and ADMA concentrations in men and women, using multiple linear regression. Both of men and women had significant and inverse associations of eGFR with ADMA plasma concentrations regardless of adjustments for potential confounders.

**Table 4:** Association of eGFR with ADMA concentrations in men and women

Variables	model 1		model 2		model 3	
	β (μmol/L)	*p* value	β (μmol/L)	*p* value	β (μmol/L)	*p* value
Men (*n* = 269)						
eGFR (mL/min/1.73m^2^)	− 0.0020	<.0001	−0.0018	0.001	−0.0015	0.005
Systolic BP (mmHg)			0.0000	0.955	−0.0001	0.746
Diastolic BP (mmHg)			−0.0009	0.292	−0.0004	0.635
Total cholesterol (mg/dL)			−0.0001	0.524	−0.0001	0.539
HDL cholesterol (mg/dL)			0.0005	0.333	0.0005	0.351
Diabetes Mellitus			0.0110	0.461	0.0130	0.393
Current smokers (for non-smokers)			0.0227	0.010	0.0224	0.014
Current drinkers (for non-drinkers)			−0.0090	0.520	−0.0079	0.574
Age (yr)					0.0015	0.210
BSA					−0.0003	0.991
Women (*n* = 382)						
eGFR (mL/min/1.73m^2^)	−0.0009	0.036	−0.0008	0.052	−0.0010	0.039
Systolic BP (mmHg)			−0.0001	0.691	0.0000	0.894
Diastolic BP (mmHg)			0.0004	0.521	0.0003	0.713
Total cholesterol (mg/dL)			0.0000	0.884	0.0000	0.895
HDL cholesterol (mg/dL)			−0.0005	0.363	−0.0004	0.434
Diabetes Mellitus			−0.0112	0.450	−0.0124	0.409
Current smokers (for non-smokers)			0.0231	0.259	0.0238	0.247
Current drinkers (for non-drinkers)			−0.0444	0.067	−0.0442	0.071
Age (yr)					−0.0005	0.608
BSA					0.0102	0.734

eGFR, estimated glomerular filtration rate; BP, blood pressure; HDL, high-density lipoprotein; BSA, body surface area

Model 1: unadjusted

Model 2: adjusted for blood pressure, total and HDL cholesterol, diabetes mellitus, smoking status, and drinking status

Model 3: adjusted for sex, age, BSA, blood pressure, total and HDL cholesterol, diabetes mellitus, smoking status, and drinking status

## Discussion

We observed a significant independent association between eGFR and plasma ADMA concentrations in the elderly Korean population. We suggested that eGFR decline might be a risk factor of endothelial dysfunction. Even for the apparently healthy elderly with no known kidney disease, reduced kidney function could predict the increased risk of endothelial dysfunction.

Our results are consistent with the findings from previous studies [14–19]. According to a prospective study, which was conducted for 227 patients with mild to moderate kidney disease, ADMA concentrations were significantly and negatively correlated with GFR, and positively correlated with age and serum creatinine. Mean ADMA concentrations in advanced chronic kidney disease (CKD) patients with GFR < 30 ml/min/1.73 m^2^ were significantly higher than in CKD patients with GFR ≥ 90 ml/min/1.73 m^2^. Furthermore, this study suggested that ADMA elevation was one of the factors that promotes the progression of CKD [14]. A cross-sectional study in Australia was conducted for 145 patients aged 40 to 74 with coronary artery disease. Although this study included only patients with GFR ≥ 45 ml/min/1.73 m^2^, patients in the low GFR group (GFR < 81 ml/min/1.73 m^2^) had significantly higher ADMA concentrations, compared to patients in the high GFR group (GFR ≥ 81 ml/min/1.73 m^2^). The association of GFR and ADMA concentrations was also independent of sex, age, and cigarette smoking habit [15].

In a subsequent Austrian study, patients with stage 4–5 CKD (eGFR < 30 ml/min/1.73 m^2^) had significantly higher ADMA concentrations than those with stage 2–3 CKD (eGFR ≥30 ml/min/1.73 m^2^). They also observed an increase in plasma ADMA concentrations and a decrease in ADMA urinary excretion in patients with stage 4–5 CKD. Therefore, they suggested that reduced eGFR might influence the high accumulation of plasma ADMA concentrations and low excretion of ADMA in urine [16]. According to a previous study which was conducted for 218 general and diabetic hypertensive patients, ADMA concentrations were inversely correlated with eGFR. They found that increases in ADMA concentrations during the follow-up period were significantly associated with declines in eGFR and the progression of CKD [17]. A previous paper observed the differences in mean ADMA concentrations among renal patients. In the results, CKD and dialysis patients had significantly higher ADMA compared to the control group [18]. One cross-sectional study consisting of Ghanaian patients with type 2 diabetes determined that there was a significant association between ADMA values and eGFR. Furthermore, this study considered ADMA level as a novel biomarker of renal dysfunction. They also suggested that a reduced eGFR in diabetic patients leads to an increase in ADMA concentrations [19].

Since our study targeted the elderly, old-age might be a factor that impacted the association between reduced kidney function and elevated ADMA concentrations. According to a paper on clinical guidelines of CKD, a decline in eGFR in the elderly is an independent predictor for adverse outcomes because eGFR considers age, sex, and body size in the equation. This study also suggested that eGFR is the best measure of kidney function [13].

This study has some limitations. First, we could not directly assess GFR. There are several ways to measure GFR directly, such as using plasma clearance of nonradioactive iohexol [20] and urinary clearance of inulin or iothalamate [21]. However, because our cohort study was conducted for the general healthy population and not for patients with suspicious kidney disease, we estimated kidney function from the CKD-EPI by using only two variables known, blood urea nitrogen and creatinine concentrations. Second, we did not assess endothelial function directly. Plasma ADMA concentration was used as a marker of endothelial dysfunction in this study. Although the mechanism of causality between ADMA concentration and endothelial dysfunction is not clear, previous evidence supports its existence [4–6]. Third, serum creatinine concentrations were analyzed by a colorimetric Jaffe, Alkaline picrate, kinetic method, but not standardized by an isotope dilution mass spectrometry (IDMS) method. IDMS is a technique with proven high accuracy for which the sources of error are understood and under control [22]. Since 2017, the IDMS method has been utilized to measure serum creatinine in Seoul Clinical Laboratories, which is a research center analyzing blood, urine, DNA, and other human data. Therefore, our kidney data was standardized by a calibrator using the high-performance liquid chromatography (HPLC) candidate reference method at 2012. Lastly, due to the cross-sectional study design, we only suggest an association between eGFR and ADMA concentration. We could not explain a causal relationship and the clear mechanisms of eGFR and ADMA concentrations. Further research should be carried out to determine the mechanisms.

Although most previous studies have been conducted for CKD patients, this study did find a significant association in general healthy older adults. Thus, we were able to assess the effect of reduced eGFR on elevated ADMA plasma concentration in the general elderly population.

## Conclusion

Our findings suggest that eGFR reduction may be related to elevated ADMA plasma concentrations in the elderly Korean population. Further studies are required to verify the prospective causal effect of eGFR reduction on ADMA concentrations in the general population.

## Data Availability

The datasets generated and/or analyzed during the current study are not publicly available due to the ethics approval for this study. However, the data are available from the corresponding author on reasonable request.

## Abbreviations

ADMA: Asymmetric Dimethylarginine
ANOVA: Analysis of Variance
BMI: Body Mass Index
BSA: Body Surface Area
CKD: Chronic Kidney Disease
CKD-EPI: Chronic Kidney Disease- Epidemiology Collaboration Group
DBP: Diastolic Blood Pressure
eGFR: estimated Glomerular Filtration Rate
HDL: High-Density Lipoprotein
SBP: Systolic Blood Pressure
